# Consistency and variability in human performance during simulate infant CPR: a reliability study

**DOI:** 10.1186/s13049-020-00785-y

**Published:** 2020-09-10

**Authors:** Debora Almeida, Carol Clark, Michael Jones, Phillip McConnell, Jonathan Williams

**Affiliations:** 1 Faculty of Health and Social Sciences, Bournemouth University, R604, Royal London House, Christchurch Road, Bournemouth, BH1 3LT England; 2 Department of Anesthesiology, Main Theatres, Royal Bournemouth and Christchurch Hospitals, Castle Lane East, Bournemouth, BH7 7DW England; 3 Faculty of Health and Social Sciences, Bournemouth University, R612, Royal London House, Christchurch Road, Bournemouth, BH1 3LT England; 4 Cardiff School of Engineering, Cardiff University, Cardiff, CF23 3AA Wales; 5 Resuscitation Services, Heart Club, Royal Bournemouth Hospital, Castle Lane East, Bournemouth, BH7 7DW England; 6 Faculty of Health and Social Sciences, Bournemouth University, R611, Royal London House, Christchurch Road, Bournemouth, BH1 3LT England

**Keywords:** Infant cardiopulmonary resuscitation, Repeatability, Reliability, Variation, Minimal detectable change

## Abstract

**Background:**

Positive outcomes from infant cardiac arrest depend on the effective delivery of resuscitation techniques, including good quality infant cardiopulmonary resuscitation (iCPR) However, it has been established that iCPR skills decay within weeks or months after training. It is not known if the change in performance should be considered true change or inconsistent performance. The aim of this study was to investigate consistency and variability in human performance during iCPR.

**Methods:**

An experimental, prospective, observational study conducted within a university setting with 27 healthcare students (mean (SD) age 32.6 (11.6) years, 74.1% female). On completion of paediatric basic life support (BLS) training, participants performed three trials of 2-min iCPR on a modified infant manikin on two occasions (immediately after training and after 1 week), where performance data were captured. Main outcome measures were within-day and between-day repeated measures reliability estimates, determined using Intraclass Correlation Coefficients (ICCs), Standard Error of Measurement (SEM) and Minimal Detectable Change (MDC_95%_) for chest compression rate, chest compression depth, residual leaning and duty cycle along with the conversion of these into quality indices according to international guidelines.

**Results:**

A high degree of reliability was found for within-day and between-day for each variable with good to excellent ICCs and narrow confidence intervals. SEM values were low, demonstrating excellent consistency in repeated performance. Within-day MDC values were low for chest compression depth and chest compression rate (6 and 9%) and higher for duty cycle (15%) and residual leaning (22%). Between-day MDC values were low for chest compression depth and chest compression rate (3 and 7%) and higher for duty cycle (21%) and residual leaning (22%). Reliability reduced when metrics were transformed in quality indices.

**Conclusion:**

iCPR skills are highly repeatable and consistent, demonstrating that changes in performance after training can be considered skill decay. However, when the metrics are transformed in quality indices, large changes are required to be confident of real change.

## Background

Cardiac arrest is a worldwide health problem associated with considerable morbidity, mortality and extensive healthcare costs [1–3]. In the UK, over 30,000 out-of-hospital cardiac arrests (OHCA) occur yearly, with an estimate of around 6000 cases in the paediatric population, and infants comprising the majority of these occurrences [4–9]. Reported survival to discharge rates range between 2 and 18% for both OHCA and in-hospital cardiac arrest and paediatric cases are associated with undesirable high-rates of both mortality and morbidity, making cardiac arrest in the infant population, a substantial public health problem [5–10].

Positive outcomes from infant cardiac arrest depend on, in part, the effective delivery of resuscitation techniques, including quality infant cardiopulmonary resuscitation (iCPR), which is crucial for perfusion of vital organs [11, 12]. Quality iCPR is dependent on achieving four internationally recommended quality measures: chest compression depth; chest compression rate; complete chest recoil; and appropriate compression duty cycle, (the portion of time spent in compression) [13–15]. However, it has been demonstrated that the quality of chest compressions during paediatric CPR (including infant) delivered by lay persons, basic life support (BLS) and highly-trained-rescuers in both simulated and real paediatric cardiac arrest events is often performed inadequately, incorrectly, inconsistently or with excessive interruption [15–18].

The cause of CPR quality is likely multifactorial, however key elements include initial skill acquisition and subsequent retention and decay of skills. Several studies have established an urgent need to identify the frequency of iCPR re-training in order to maintain adequate and effective skills [19–23]. However, there are no studies that have examined the consistency of human performance during CPR and this study aims to address this knowledge gap.

Establishing variability in human performance is critical to understanding changes and determining natural variations in the delivery of a skill like iCPR. For example, if performance is not consistent within days of training, then any differences seen months later could be due to variation and inconsistent performance rather than skill decay. This is critical for the determination of skill decay which will facilitate the optimization of training intervals for CPR. Currently, no data has been reported on individuals’ iCPR performance variability. Therefore, the aim of this study is to determine the repeated measures reliability and variability of individuals performing simulated iCPR.

## Methods

### Study design and setting

An experimental, prospective, observational design was used to test within-day and between-day reliability of iCPR performance and was conducted within a university setting. Bournemouth University Research Ethics Committee approval was obtained (reference ID: 22558) and following explanation of experimental procedures, written informed consent was gained. Age, sex, height, weight and self-declared physical issues that might compromise performance were gathered in order to create a demographic profile of the sample.

### Participants

A convenience sample of 27 participants were recruited from university healthcare students (including Operating Department Practice, Physiotherapy, Nursing, Midwifery and Occupational Therapy). Inclusion criteria: students currently enrolled at the university with no previous training in paediatric life support. Exclusion criteria: students with any form of musculoskeletal pain requiring medical intervention in the last 12 months or self-declared inability to physically perform iCPR. Sample size was calculated based on Walter et al. (1988) [24] with alpha = 0.05; beta = 80%; three repetitions of the task; desirable and minimal correlation values set at 0.8 and 0.6 respectively, yielding a necessary sample size of 27.

### Study procedures

On completion of informed consent, participants undertook the standard four stage approach ‘Paediatric BLS’ education package [25] with compression:ventilation ratio of 30:2 (aligned with resuscitation guidelines for BLS rescuers with no duty to respond to a paediatric cardiac arrest), delivered by a qualified instructor (PM). After 30-min practice on an infant manikin (Laerdal® ALS Baby, Laerdal Medical, Stavanger, Norway) using the 2-finger technique, participants were invited to a separate room set up with the instrumented manikin (description below). Although the researcher was in the same room as the participants during data collection to initiate and pause the software, apart from brief instructions on when to start and stop the iCPR, there was no further interaction between participants and the researcher during data collection, and the participants didn’t receive any form of feedback. Performance data pertaining to iCPR was captured on two occasions, firstly immediately following training and secondly after 1 week. This timeframe was deliberately selected to potentially avoid skill decay, which has been demonstrated to occur within weeks to months after training [21, 26]. Skill decay would interfere with the reliability results of this study therefore, a pragmatic 1-week interval has been selected. On each occasion, 3 trials of 2-min iCPR were captured, with 1-min interval between trials.

### Equipment

The equipment used to quantify iCPR performance comprised of: (a) a CPR infant manikin (Laerdal® ALS Baby, Laerdal Medical, Stavanger, Norway) representing a three-month-old, 5 kg infant. This manikin was modified during a previous study and its consistency has been established [27]. The modification was an improvement to allow the maximum compression depth to vary between 40 mm (original manikin specification) up to 56 mm (physiological internal chest depth of a three-month-old infant).; (b) 2 accelerometers; (c) data acquisition unit; (d) personal computer; (e) flow sensor and (f) power supply.

One accelerometer was fixed over the xiphoid process on the manikin’s chest and the other used to act as a differential for the ‘surface’ on which the CPR was conducted, the floor in our case to mimic the conditions of OHCA.

The LabView software platform was used to power the accelerometers and compute double integrated acceleration data to provide chest displacement. Validity of this displacement data has previously been established [28]. Displacement data were transferred to MATLAB 2008b (The MathWorks Inc., Natick, MA) where a bespoke algorithm converted it into four metrics, average compression depth, average compression rate, average residual leaning and average duty cycle. Compression depth was defined as the maximum relative displacement between the two accelerometers and compression rate as the number of compressions per minute. Residual leaning was determined through incomplete release from the chest wall measured in mm and converted to kg through the known stiffness of the manikin. Duty cycle was defined as the ratio of time taken for compression relative to release and was calculated using a new algorithm, as published previously [29]. These metrics were further converted into quality indices (QI) by determining the percentage of compressions which met European Resuscitation Council Paediatric (Infant) Life Support Guidelines for Resuscitation (2015) [30] and Resuscitation Council UK Paediatric (Infant) BLS guidelines (2015) [14] outlined below.

### Outcome measures

Primary outcomes - degree of correlation between repeated measures for: (i) chest compression rate, (ii) chest compression depth, (iii) residual leaning, and (iv) duty cycle, to establish reliability and consistency of iCPR performance.

Secondary outcomes - degree of correlation between repeated measures for each respective quality indices (QI) defined as: (i) compression rate (100–120/min), (ii) compression depth (40 mm - 45 mm),, (iii) residual leaning (<2.5 kg) and (iv) duty cycle (45–50%).

### Statistical analysis

Demographic data were analyzed using descriptive statistics. Mean (SD) were used to report the data with a normal distribution, and median [IQR] were used when the assumption of normality was not met via Skewness, Kurtosis and Shapiro-Wilk test. Both within-day and between-day repeated measures reliability for each variable was determined using Intraclass Correlation Coefficient (ICC) estimates and their 95% confidence intervals (*Model:* 2-way mixed effects; *Type:* multiple measurements; *Definition:* absolute agreement), with values less than 0.5 indicating poor reliability, between 0.5 and 0.75 moderate reliability, between 0.75 and 0.9 good reliability, and greater than 0.90 excellent reliability [31]. Standard Error of Measurement (SEM) was also calculated to report variability of results in the units of interest and Minimal Detectable Change (MDC) was calculated to quantify the natural variation in performance, using the following equation: MDC_95%_ = 1.96 x SEM x √2.

Microsoft Office Excel 2016 (Microsoft Corporation) and IBM SPSS Statistics version 25 (IBM Corp., Armonk, NY, USA) were used for statistical calculations.

## Results

### Participants demographics

A total of 27 healthcare students participated in this study including 20 females (74.1%). The mean (SD) age was 32.6 (11.6) years; height was 1.7 (0.1) meters and weight was 70.9 (12.4) kg. Each participant had previously received adult BLS training. No participants were lost at follow up.

### Within-day reliability - chest compression variables

The mean values, ICC, SEM and MDC, are presented in Table 1.

**Table 1:** Mean chest compression and within-day reliability - Time point 1

	Mean (SD)	ICC (95% CI)	SEM	MDC	MDC as % of mean
**Rate (cpm)**	104 (16)	.95 (.91–.97)	3.47	9.61	9%
**Depth (mm)**	42.9 (4.7)	.96 (.92–.98)	0.95	2.64	6%
**Leaning (kg)**	2.9 (0.9)	.94 (.88–.97)	0.23	0.64	22%
**Duty cycle (%)**	44 (11)	.95 (.91–.98)	2.41	6.68	15%

*cpm* Compressions per minute, *SD* Standard deviation, *ICC* Intraclass correlation coefficient, *SEM* Standard error of measurement, *MDC* Minimal detectable change

A high degree of reliability was found for repeated iCPR for every variable with excellent ICCs and narrow confidence intervals. SEM values were low, demonstrating excellent consistency in repeated within-day performance.

MDC values were low for compression depth and compression rate (6 and 9%) and slightly higher for duty cycle (15%) and residual leaning (22%).

### Between-day reliability – chest compression variables

The ICC, SEM and MDC for between-day reliability (immediately after training and 1 week later) and absolute difference between means for the two time points are presented in Table 2.

**Table 2:** Mean absolute difference chest compression and between-day reliability – Time point 1 and Time point 2

	Mean (SD) absolute difference	ICC (95% CI)	SEM	MDC	MDC as % of mean (time point 1)
**Rate (cpm)**	9.0 (7.0)	.86 (.69–.94)	2.63	7.28	7%
**Depth (mm)**	1.9 (1.6)	.92 (.68–.97)	0.44	1.23	3%
**Leaning (kg)**	0.6 (0.5)	.78 (.42–.91)	0.23	0.65	22%
**Duty cycle (%)**	5.7 (6.7)	.75 (.44–.89)	3.38	9.36	21%

*cpm* Compressions per minute, *SD* Standard deviation, *ICC* Intraclass correlation coefficient, *SEM* Standard error of measurement, *MDC* Minimal detectable change

The ICC values ranged from good to excellent, with duty cycle demonstrating the lowest ICC and compression depth the greatest. The SEMs were low suggesting good consistency between days. The percentage MDC for between days followed the same pattern as within-day, with low values for compression depth and compression rate (3 and 7%) and higher for duty cycle (21%) and residual leaning (22%).

### Within-day reliability - chest compression quality indices (QI)

The mean values, ICC, SEM and MDC for each QI are presented in Table 3.

**Table 3:** Mean chest compression QI and within-day reliability for QI - Time point 1

	Mean (SD)	ICC (95% CI)	SEM	MDC	MDC as % of mean
**Rate QI (%)**	38 (29)	.90 (.81–.95)	9.26	25.67	67%
**Depth QI (%)**	45 (19)	.91 (.83–.96)	5.64	15.63	35%
**Leaning QI (%)**	42 (21)	.94 (.88–.97)	5.23	14.49	34%
**Duty cycle QI (%)**	18 (17)	.87 (.76–.94)	6.03	16.73	93%

*SD* Standard deviation, *ICC* Intraclass correlation coefficient, *SEM* Standard error of measurement, *MDC* Minimal detectable change

The ICC values were good to excellent for each QI with small confidence intervals suggesting a high level of reliability. The MDC values were higher than the equivalent primary variables, suggesting greater variability in performance when measured by QI.

### Between-day reliability – chest compression quality indices (QI)

The ICC, SEM and MDC for between-day reliability using QI are presented in Table 4.

**Table 4:** Mean absolute difference chest compression QI and between-day reliability for QI - Time point 1 and Time point 2

	Mean (SD) absolute difference	ICC (95% CI)	SEM	MDC	MDC as % of mean (time point 1)
**Rate QI (%)**	21 (20)	.52(−.07–.78)	13.83	38.33	101%
**Depth QI (%)**	12 (9)	.81(.54–.91)	3.38	9.33	21%
**Leaning QI (%)**	15 (13)	.77(.36–.90)	6.30	17.47	42%
**Duty cycle QI (%)**	10 (11)	.68(.29–.85)	6.25	17.33	96%

*cpm* Compressions per minute, *SD* Standard deviation, ICC Intraclass correlation coefficient, *SEM* Standard error of measurement, *MDC* Minimal detectable change

The ICC values ranged from moderate to good, with compression rate QI demonstrating the lowest value and compression depth QI the highest value.

The SEMs were low for compression depth QI, residual leaning QI and duty cycle QI suggesting good consistency between days for these variables. The percentage MDC was moderate for compression depth QI (21%), but considerably higher for the other QIs, demonstrating greater variability when measuring reliability of performance using QI.

## Discussion

The aim of this study was to determine the repeated measures reliability and variability in performance of simulated iCPR. To the authors knowledge, this is the first time such an exploration has been conducted, providing novel insights into consistency of performance. Such insights enable the determination of change above natural variability in the performance of iCPR. This is an important area of inquiry related to the design of resuscitation training and interpretation of data on skill decay.

This study makes a number of additions to the existing literature. Firstly, the results suggest that the within-day reliability (straight after training) was good-to-excellent across each of the metrics considered. Little difference was determined between each of the variables suggesting no one metric was more reliable than the other. The MDC values, however, suggest that changes greater than 22% of the mean, are likely to be greater than that witnessed through natural variation for residual leaning and 15% for duty cycle. This indicates that these two determinants of performance are less consistent, thus requiring greater change to be considered true change.

Secondly, the results demonstrate similar findings were evident for between-day reliability where values were good to excellent. Chest compression rate and chest compression depth demonstrated higher ICC values with residual leaning and duty cycle achieving good ICC values. This remains evident when comparing MDC; with compression rate and compression depth presenting very small MDC values but residual leaning and duty cycle presenting 3 times the normalized MDC, suggesting much greater variability in these two variables. Previous studies haven’t explored the ICC or MDC, making comparison to the literature difficult and this study’s contribution to the existing knowledge novel.

There are a number of possibilities to explain why there may be greater variability in these two metrics. It is possible that these aspects of performance are less well known and understood by participants. The idea of rate and depth have been the subject of many media campaigns and even without training individuals are likely to understand the importance of these two metrics, with little or no attention being placed on leaning or duty cycle. Furthermore, during the standard training for basic life support, less attention is again drawn to these two variables. Therefore, participants are less likely to be concentrating on these particular aspects of performance. Moreover, it is possible that these concepts are more difficult to learn.

It is well understood that in the initial learning phase, there is a reduction in variability towards mastery [32]. Perhaps, the results are merely a reflection of immature learning or novice performance of the motor task, which is critical for effective reduction of variability associated with leaning and duty cycle.

Another point to be considered when analyzing the reasons of greater variability in residual leaning and duty cycle is that, as previously suggested, there may be interactions between different metrics of CPR [33]. It was determined that a faster compression phase and slower relaxation phase, which produces a shorter duty cycle, correlated with a deeper compression depth. Therefore, small variations in compression depth will also be born out as variance in other metrics due to the relationship between them.

It is common place to convert actual numbers denoting CPR performance into quality indices or composite variables [16, 20, 22, 23, 34, 35]. This provides the reader with an understanding of the context pertaining to what is to be considered ‘good quality’ CPR. Despite this, the present study is the first to explore the notion of reliability of such a method of quantifying iCPR performance, therefore, filling this gap in the literature. Therefore, the third contribution made by this study is the demonstration that reliability was lower, and variability higher once iCPR variables are converted into such quality indices. The within-day reliability values remain good to excellent with small confidence intervals, however, due to much larger standard deviations, the SEM values and MDC values are much greater. This suggests that converting the values on individual metrics into the dichotomy of ‘good quality CPR’ or ‘bad quality CPR’ seems to result in greater variance in measured performance. Perhaps, this is due to individuals being close to the boundary of good/bad performance (i.e. around 50% for duty cycle). If some individuals near the 50% boundary, occasionally produce a ‘good quality’ compression/relaxation percentage and sometimes a ‘bad quality’ one, then the overall mean of them might be somewhere near the 50%. However, the duty cycle QI could be quite varied across those individuals. This may drive the variance witnessed for the quality indices, raising questions whether there should be a ‘hard cut off’ at the quality boundaries (i.e. would 51% really be ‘bad’ but 50% ‘good’ duty cycle?).

The results presented by this study demonstrate that chest compression performance during iCPR is consistent and reliable. It is also indicated that changes in performance between days which are greater than 3% of the initial value for compression depth, 7% for compression rate, 21% for residual leaning and 22% for duty cycle, represent true change in performance, indicating a decay or improvement of the iCPR skill. Higher changes are required when those metrics are converted into quality indices. Such values can serve as a reference for the learning, maintenance, improvement and decay of the metrics associated with the skills of iCPR.

### Limitations

Our study has some limitations. First, we used an infant manikin to evaluate chest compressions quality and although various studies investigating CPR performance use both paediatric and adult manikins, it is recognized that they may not exactly replicate the characteristics and chest compliance of human beings.

Second, the data collection sessions were based on individual iCPR performance in the simulated context, and as such, the participants were not exposed to background noise, other rescuers, distractions, interruptions or the stress and complications that may occur during a real cardiac arrest, limiting the transferability of our results to real life performance.

Third, we recruited a group of students from a single institution. Therefore, the caution is advised before generalization to the wider population.

Finally, we did not include rescue breaths in the analysis of our study even though ventilation is an extremely important aspect of paediatric CPR. Our focus was on chest compressions quality and consistency and we suggest that further studies should assess rescue breath skills to fully understand the reliability and consistency of those skills during iCPR performance.

## Conclusions

In summary, our study provides important additions to the growing evidence about iCPR skill acquisition and decay. For the first time, the consistency of performance of iCPR has been explored with results demonstrating that iCPR performance was highly repeatable and consistent, and this was maintained over a week. These results provide an opportunity to further explore iCPR skill acquisition and decay. Another important addition is that, when the chest compression metrics are converted into quality indices, which is a methodology commonly used in many resuscitation studies, the results are not as consistent or repeatable and the natural variation should therefore be taken into account. This means, future studies should consider investigating both metrics and quality indices when reporting individual iCPR skills performance, in order to differentiate between natural variation and skill development or decay. Previous studies, however, have explored skill acquisition and decay based on the conversion of metrics into quality indices without an acknowledgment of natural variation, potentially resulting in erroneous conclusion.

The clinical relevance of this study is attributed to the understanding of iCPR skill acquisition, maintenance, variability and decay, which will enhance iCPR training and performance, improving the chances of survival after infant cardiac arrest.

## Data Availability

The datasets used and/or analysed during the current study are available from the corresponding author on reasonable request.

## Abbreviations

BLS: Basic life support
CPR: Cardiopulmonary resuscitation
iCPR: Infant cardiopulmonary resuscitation
ICC: Intraclass correlation coefficient
IQR: Interquartile range
MDC: Minimal detectable change
OHCA: Out-of-hospital cardiac arrests
QI: Quality index
SD: Standard deviation
SEM: Standard error of measurement
